# Large-area functionalized CVD graphene for work function matched transparent electrodes

**DOI:** 10.1038/srep16464

**Published:** 2015-11-09

**Authors:** Thomas H. Bointon, Gareth F. Jones, Adolfo De Sanctis, Ruth Hill-Pearce, Monica F. Craciun, Saverio Russo

**Affiliations:** 1 Centre for Graphene Science, College of Engineering, Mathematics and Physical Sciences, University of Exeter, Exeter EX4 4QF, United Kingdom; 2 National Physical Laboratory, Teddington TW11 0LW, United Kingdom

## Abstract

The efficiency of flexible photovoltaic and organic light emitting devices is heavily dependent on the availability of flexible and transparent conductors with at least a similar workfunction to that of Indium Tin Oxide. Here we present the first study of the work function of large area (up to 9 cm^2^) FeCl_3_ intercalated graphene grown by chemical vapour deposition on Nickel, and demonstrate values as large as 5.1 eV. Upon intercalation, a charge density per graphene layer of 5 ⋅ 10^13^ ± 5 ⋅ 10^12^ cm^−2^ is attained, making this material an attractive platform for the study of plasmonic excitations in the infrared wavelength spectrum of interest to the telecommunication industry. Finally, we demonstrate the potential of this material for flexible electronics in a transparent circuit on a polyethylene naphthalate substrate.

The development of flexible photovoltaic and light emitting devices depend on the availability and compatibility between flexible and transparent electrodes and photoactive materials. The most widely used transparent conductors nowadays -i.e. Indium-Tin-Oxide (ITO) and Fluorine doped Tin Oxide (FTO)- are brittle and their electrical properties degrade significantly under small applied strains^1^,^2^,^3^. Furthermore, the diffusion of Indium from ITO into photoactive layers of phovoltaic (PV) or organic light emitting diode (OLED) is also a well-known cause of device degradation^4^. The search for the best suitable transparent and flexible conductor is an open quest.

Meshes of metallic nano-wires are emerging as an attractive alternative, since they have high optical transmission (≈80% at 550 nm) and low sheet resistance (38Ω/◻)^5^ even when subjected to 16% strain^1^. However, scattering of light off nano-wires can introduce significant optical haze, limiting the range of applications for which these materials are suitable. Graphene is an emerging contender for enabling future flexible electronics. This single layer of sp2 hybridized carbon atoms has high optical transmission (97.6%)^6^, does not suffer of haze and the conductance is unchanged even when subject to strains up to 6.2%^7^. Contrary to ITO, no carbon migration has been reported in PV and OLED that use graphene electrodes. However, the efficiency of these devices is still low owing to the high sheet resistance of graphene (>500Ω/◻)^8^ and poor matching of the work function of graphene and that of the photoactive layer.

The sheet resistance of functionalized CVD graphene reported to date still greatly exceeds that of ITO ≈8Ω/◻ causing excessive dissipation in electrical devices^9^,^10^,^11^,^12^. The high sheet resistance sets an upper limit to the maximum transparent electrode area for example of use in OLED devices. Indeed, the potential drop across the area of a transparent electrode increases with its area, and this would result in gradients of light intensity over the surface of the OLED. Furthermore, since the work function of pristine graphene is comparable to that of ITO (4.6 eV for graphene and 4.8 eV for ITO^4^), pristine graphene electrodes require electron or hole blocking layers for efficient PVs to account for the large work function mismatch between the electrode and the photoactive layers. Changing the work function of the electrodes to match the highest occupied and lowest unoccupied molecular orbitals of the photoactive layer would minimise the barrier for charge tunnelling and reduce the energy loss of carrier thermalisation. Finding a transparent, flexible and highly conductive material that does not require charge blocking layers when embedded in PVs would enable the developement of conceptually new electronic applications.

Here we present the first experimental study of the work function (WF) of large area (up to 9 cm^2^ on glass and up to 1 cm^2^ on Si) FeCl_3_-FLG obtained from Ni-grown graphene. We find values as high as 5.1 eV, that is 0.3 eV larger than the work function of ITO, making this material an ideal replacement for ITO in PV and OLED since it would not require the additional charge blocking layer typically used with ITO electrodes. With the aid of Raman spectroscopy, we estimate a charge density per graphene layer of FeCl_3_-FLG up to 5 ⋅10^13^ ± 5⋅10^12^ cm^−2^, making this material an attractive platform for studying the physics of plasmons in the infrared region. Finally we demonstrate the potential of this material for flexible electronics in a transparent circuit on an insulating, transparent and flexible sheet of Polyethylene naphthalate (PEN) with a room-temperature resistivity of 20.52Ω/◻ and high optical transmission of 77% in the visible wavelength range.

## Results

Large-area multilayer graphene grown on nickel (Wafer of 100 mm Graphene Film on Nickel purchased from Graphene supermarket) was transferred to glass substrates by using PMMA as a support during the wet-etching of nickel in a FeCl_3_ solution. The films were subsequently transferred to ultra pure water, to a concentrated HCl solution for 1 hour and rinsed in ultra pure water^13^. Finally, the stack of multilayer graphene/PMMA was transferred to glass substrates and, after 24 hours, the PMMA was removed with acetone. A study of the optical contrast shows that 35% of this material is a four-layer with an average domain area of 150*μ*m^2^, see Supplementary information. Two separate sets of multilayer graphene samples were intercalated with FeCl_3_ for 8 and 24 hours following a previously demonstrated two-zone vapour transport method^14^,^15^. The resulting material is a heavily doped form of graphene highly stable to high levels of humidity (>95% relative humidity) and high temperature (up to 200°C in air and at least 650°C in vacuum)^16^ which was previously shown to be a good replacement for gold electrodes in photodetectors devices^17^.

Scanning Kelvin probe microscopy (SKPM) is used to determine the absolute work function (Φ) and its spatial distribution over the surface of intercalated graphene. SKPM maps the electrostatic forces between the surface of the sample and a conductive atomic force microscopy (AFM) probe which is oscillated above the sample surface. The electrostatic force between the cantilever and the sample is nullified by applying a backing dc bias to a conductive AFM probe. The applied bias which is equal and opposite to the electrostatic potential is termed the surface potential (SP) and is equivalent to the contact potential difference (V_CPD_) measured in static Kelvin probe measurements. The accuracy with which the absolute work function of a material using Kelvin probe methods can be calculated depends on the accuracy with which the work function of the probe can be determined. For these measurements we employed an NT-MDT NTEGRA Aura system using frequency-modulated SKPM mode and conductive Bruker PFQNE tips in ambient conditions.

**Figure 1:**
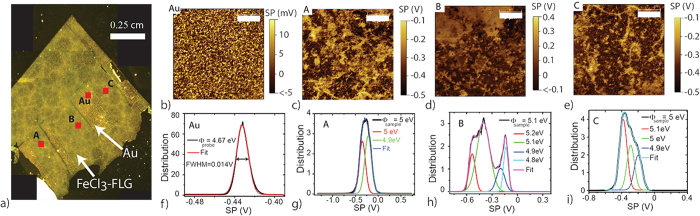
(**a**) Micrograph picture of an intercalated sample with Au contacts deposited on top. (**b**–**e**) SKPM maps of representative 20 *μ*m^2^ areas on Au and three other locations on FeCl_3_-FLG as highlighted in the panel (**a**). (**f**–**i**) Graphs of the associated distributions to the SKPM maps shown in panels (**b**–**e**) reporting the calibrated Φ_sample_. The tip was calibrated on an area of gold electrode shown in (**b**) with the corresponding distribution in (**f**). A Φ_probe_ of 4.67 eV was calculated assuming a Φ_Au_ of 5.1 eV.

Gold electrodes were deposited onto the intercalated samples serving both as a grounding contact and a calibration standard for the measurements of Φ, see Fig. 1a–i. The Φ of the AFM probe was calibrated by comparison with that of the gold electrodes; (Φ_probe_ = Φ_Au_ + V_CPD_) where Φ_Au_ is assumed to be 5.1 eV^18^. Gold is chosen as a standard in Kelvin probe measurements as it does not oxidise or readily undergo chemical reactions. A map of the spatial dependence of the SP of the gold film is shown in Fig. 1b. The corresponding SP distribution is found to be narrow demonstrating the homogeneity of Φ_Au_ with an average SP of −0.43 V and a full width at half maximum (FWHM) of 0.014 eV giving a Φ_probe_ of 4.67 eV ± 8 meV, see Fig. 1f. Figure 1b–c,g–i show SKPM maps and SP distribution histograms for representative 20 *μ*m^2^ areas of the intercalated sample highlighted in the micrograph picture of Fig. 1a. Several distinct SPs are observed in the SKPM maps with distribution histograms showing overlapping peaks corresponding to a distribution of Φ_sample_. The most commonly occurring Φ_sample_ were recorded at 5.1, 5 and, 4.9 eV with the majority of the surface area of the sample ≈60% showing a work function value as large as 5.1 eV (see Supporting information). The observed three dominant values of Φ_sample_ are to be expected in intercalated few-layer graphene and correspond to the following three distinct cases: (1) graphene sandwiched between FeCl_3_, (2) graphene with FeCl_3_ only on one side and (3) graphene with no direct contact to FeCl_3_. While the SP was observed to vary on the nanoscale between 4.8–5.2 eV, the average Φ_sample_ value of 5 eV was uniform over the scale of the sample -that is 1 cm^2^.

**Figure 2:**
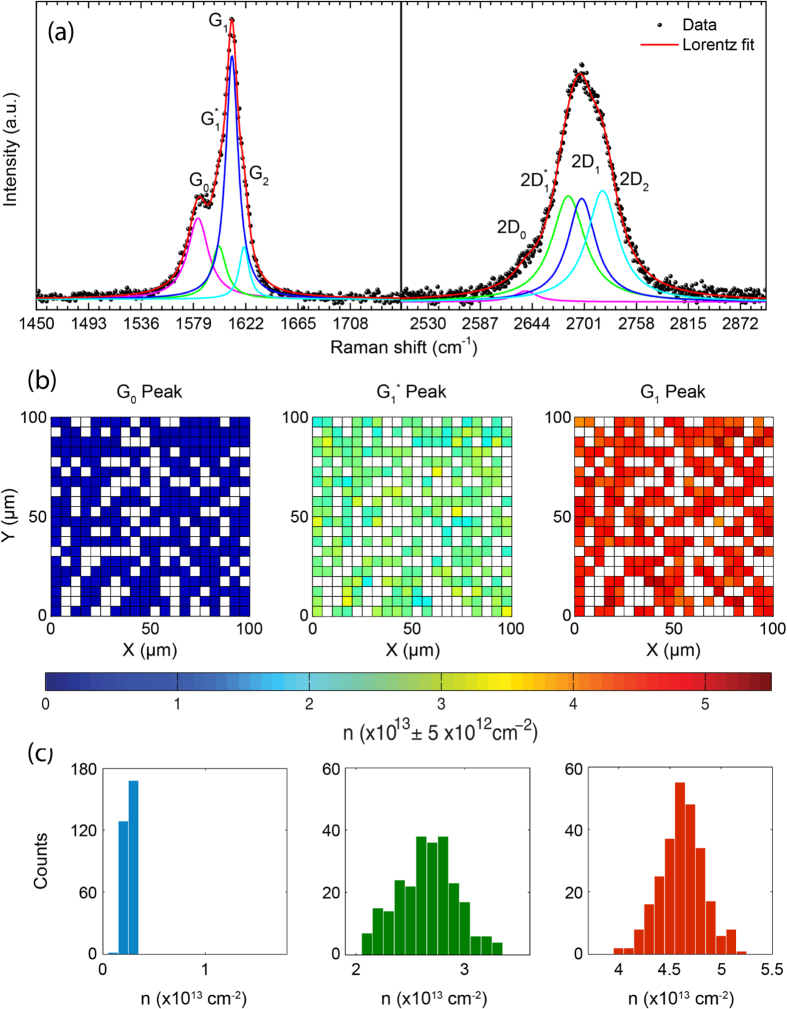
(**a**) Representative Raman spectrum (532 *nm* laser excitation) of large-area FeCl_3_ intercalated few-layer graphene showing the G- (1580–1630 cm^−1^) and 2D-band regions (≈2700 cm^−1^) with the respective four Lorentzian peaks fit (G_0_-2D_0_, 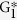-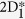, G_1_-2D_1_ and G_2_-2D_2_). (**b**) Spatial distribution of the hole concentration for the first three intercalation stages, over an area of 100 × 100 *μ*m^2^ mapped in steps of 5 *μ*m. (**c**) Statistical study of *n* for each intercalation stage over the 400 points in (**b**).

Raman spectroscopy is another valuable tool for the characterization of this material. Figure 2a shows a representative Raman spectra of a 1 cm^2^ multilayer graphene on a Si/SiO_2_ substrate intercalated for 36 h. We find that the G-band (1580–1630 cm^−1^) is composed by four Lorentzian peaks labelled as G_0_ (1580 cm^−1^), 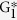 (1600 cm^−1^), G_1_ (1611 cm^−1^) and G_2_ (1622 cm^−1^). Each G-peak corresponds to a different level of charge transfer^14^,^19^,^20^. Upon doping the 2D-band (≈2700 cm^−1^) is upshifted and its shape becomes the convolution of a smaller number of Lorentzians as compared to the pristine multilayer graphene^21^. We find that four Lorentzians are needed to obtain a good fit to the 2D-band shown in Fig. 2a with maxima at 2637 cm^−1^ (2D_0_), 2684 cm^−1^ 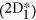, 2700 cm^−1^ (2D_1_) and 2721 cm^−1^ (2D_2_).

The positions of G- and 2D- peaks are unique identifiers of the doping level in this intercalated compound. More specifically, G_0_ and 2D_0_ are characteristic of un-doped (pristine) graphene layers. G_1_ and 2D_1_ indicate that graphene is in direct contact to only one adjacent FeCl_3_ layer (low doping), whereas G_2_ and 2D_2_ appear when graphene is sandwiched between two FeCl_3_ layers (high doping)^14^. 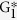 and 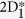 is attributed to graphene in direct contact to only one adjacent FeCl_3_ layer but with a lower density of FeCl_3_ molecules, thus giving lower charge transfer.

## Discussion

To characterize the Fermi energy in FeCl_3_-FLG from the Raman spectra we use the model developed by Lazzeri *et al*.^22^ where doping of graphene stiffens the E_2*g*_ phonon mode of the Raman G-peak due to the non-adiabatic removal of the Kohn anomaly at Γ^23^. Within this model, a unique relation between the charge density *n* and the upshift of the G-peak position was obtained and this can efficiently be used to determine *n* with an accuracy better than 10% from the values of charge density obtained with complementary methods such as quantum oscillations of magneto-conductance, see Supplementary information. Estimates of *n* over a representative surface area of 10^4^ *μm*^2^ in our material show three different doping levels, see Fig. 2b. The presence of three distinct G-peaks in the Raman spectra allow us to extrapolate three corresponding charge concentrations: *n*_0_ = 0.3 ⋅ 10^13^ ± 1 ⋅ 10^12^ cm^−2^, 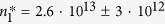 cm^−2^ and *n*_1_ = 4.6⋅10^13^ ± 5 ⋅ 10^12^ cm^−2^, see Fig. 2c. In a FeCl_3_-intercalated multilayer graphene system, such as Ni-grown CVD graphene, the total charge density can reach values as high as 10^15^ cm^−2^ for a sequence of 15 graphene layers.

Since the exploitation of collective charge oscillations in graphene (i.e. plasmons) for light manipulation strongly relies on doping, FeCl_3_-intercalation of multilayer graphene provides an attractive platform for pioneering studies of surface plasmons at wavelengths of interest to the telecommunication industry. More specifically, the plasmon frequency in graphene scales as *ħω* ∝ (*E*_*F*_/*D*)^1/2^ where *E*_*F*_ is the Fermi level and *D* is the size of the resonant plasmonic structure^24^. A Fermi energy of *E*_*F*_ ≈ 1 *eV*, corresponding to a total charge density of ≈7 ⋅ 10^14^ cm^−2^, would give a plasmon resonance at a photon wavelegth of ~1.4*μ*m^24^. Such a high charge density can be easily attained in FeCl_3_-intercalated multilayer graphene.

**Figure 3:**
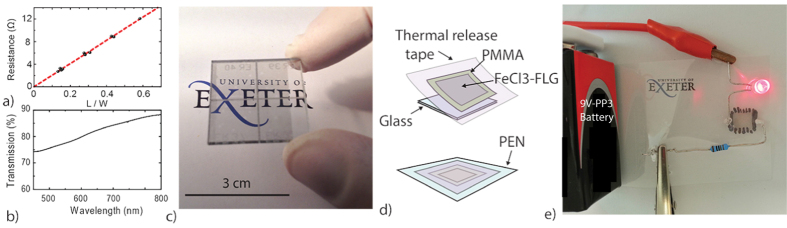
(**a**) Plot of the resistance as a function of the FeCl_3_-FLG channel aspect ratio defined as length over width (L/W). (**b**) Plot of the optical transmittance in the visible wavelength range. The experimental error in transmittance is smaller than 0.1%. (**c**) Image of large-area FeCl3-FLG on a 9 cm^2^ glass substrate made using a PMMA-assisted wet transfer technique. (**d**) Schematic representations of the dry transfer method used to transfer FeCl_3_-FLG from a glass substrate to the target flexible and transparent PEN substrate. (**e**) Picture of an electric circuit which uses the FeCl_3_-FLG on PEN as an electric line to light up a red LED.

The room temperature resistivity of the Ni-grown intercalated multilayer graphene was characterized by measuring the four-terminal resistance in devices with different aspect ratios of the conductive channel, that is device length -ranging from 0.15 cm to 0.7 cm- divided by the device width (1 cm), see Fig. 3a. A linear fit to these measurements gives a typical sample resistivity of 20.52 ± 0.48Ω/◻, which is ≈1000 times smaller than the resistivity of graphene at the neutrality point and more than 20 times smaller than the lowest values of resistivity reported in CVD-grown graphene to date. At the same time we find that the optical transmittance (T) of intercalated multi-layer graphene on a glass substrate in the visible wavelength range from 450 nm to 850 nm is larger than 74%, see Fig. 3b,c. In particular, at 550 nm T is higher than 77%, and this is comparable to the value for a 10*μ*m film of ITO of 80%^12^. Upon increasing the wavelength to 850 nm the optical transmission increases monotonously to 87%.

The unique combination of (1) large work function, (2) low electrical resistivity and (3) high optical transmittance make this material an attractive system for flexible electronics and PV applications. To demonstrate further its potential, we have transferred the intercalated Ni-graphene to a standard flexible PEN substrate. This is readily done by spin coating PMMA onto FeCl_3_-FLG and using a thermal release tape to mechanically peal the functionalized graphene off the glass substrate see Fig. 2c. Subsequently, the large area FeCl_3_-FLG of 1 cm^2^ was dry transferred to a PEN substrate, while the tape and PMMA are removed by a heat process and rinsing in acetone, respectively (see Fig. 3d). We then demonstrate the use of FeCl_3_-FLG as a transparent conductor by closing the circuit of a battery lighting up an LED through this functionalized graphene, as shown in Fig. 3e. Note that owing to the high electrical conductivity of this material, it is necessary to introduce a resistor in series to the circuit to limit the voltage drop across the LED to less than 2 V.

In conclusion we have presented the first systematic study of the work function of large area FeCl_3_-FLG obtained from Ni-grown graphene (up to 9 cm^2^ on glass and 1 cm^2^ on Si and PEN), with values as large as 5.1 eV, which qualify this material as a replacement for ITO for example in PV and OLED. Upon intercalation, a charge density per graphene layer of ≈5 ⋅ 10^13^ cm^−2^ is attained, making this material an attractive platform for the study of plasmons in the infrared region potentially of interest to the telecommunication industry. Finally, we demonstrate the potential of this material for flexible electronics in a transparent circuit on a PEN substrate with a room temperature resistivity of 20.52Ω/◻ and optical transmission of 77% in the visible wavelength range.

## Additional Information

**How to cite this article**: Bointon, T. H. *et al*. Large-area functionalized CVD graphene for work function matched transparent electrodes. *Sci. Rep.* **5**, 16464; doi: 10.1038/srep16464 (2015).

## Supplementary Material

Supplementary Information
