# Supplementary material for: Large-area functionalized CVD graphene for work function matched transparent electrodes
Source: Sci Rep. 2015 Nov 9;5:16464. doi: 10.1038/srep16464 (PMC4997100; doi:10.1038/srep16464)
Supplement: Supplementary Information [file srep16464-s1.pdf]

Supporting Information

Large-area functionalized CVD graphene for work function matched transparent electrodes

Thomas H. Bointon[1], Gareth F. Jones[1], Adolfo De Sanctis[1], Ruth Hill-Pearce[2], Monica F. Craciun[1], and Saverio Russo[1]*

1 Centre for Graphene Science, College of Engineering, Mathematics and Physical Sciences, University of Exeter, Exeter EX4 4QF, United Kingdom
2 National Physical Laboratory, Teddington TW11 0LW, United Kingdom

E-mail: S. Russo@exeter.ac.uk

1. Spatial dependence of the work function

To ascertain the spatial distribution of the work function (Φ) we have conducted a statistical study of the estimated values of Φ in three representative areas of about 20 μm^2 highlighted in the micrograph picture of Figure S1. The most commonly occurring values of work function recorded at 5.1, 5 and 4.9 eV with the majority of the surface area of the sample about 60% characterized by a work function value of 5.1 eV (see Table S1). The observed three dominant values of work function are consistent with three distinct cases: graphene sandwiched between $FeCl_3$, graphene with $FeCl_3$ only on one side and graphene with no direct contact to $FeCl_3$.

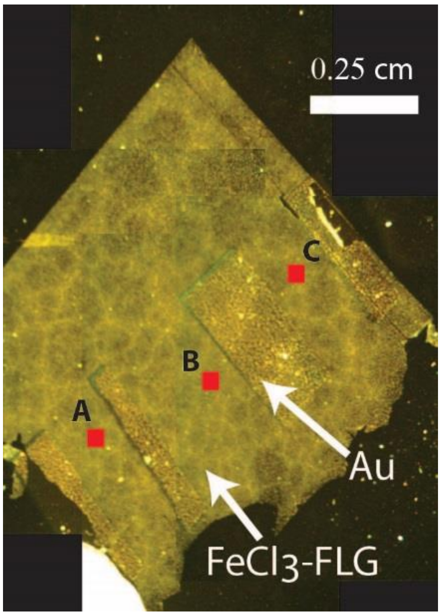

Figure S1. Micrograph picture of an intercalated sample with Au contacts deposited on top.

Table S1. Percentage coverage of the most commonly observed Φ_{sample} in the three studied areas indicated in Figure S1.

Area	Φ = 4.9 eV	Φ = 5 eV	Φ = 5.1 eV
A	45%	55%	-
B	24%	29%	46%
C	13%	-	59%

2. Characterization of Ni-grown CVD graphene

Nickel-grown CVD graphene purchased from Graphene-supermarket (Wafer of 100mm Graphene Film on Nickel) was used in this work since this growth method gives multilayer graphene on large areas (up to 100 cm^2) which is needed for the intercalation of $FeCl_3$ [3]. The product datasheet specify that this is a continuous film of multilayers (up to 7-10 layers). The characterization of the multilayer graphene transferred on Si/SiO_2 (with 300nm thick SiO_2) substrate is shown in Figure S2. More specifically, Figure S2a shows a false-colour map of an optical micrograph picture (see Figure S2b) of transferred multilayer graphene on Si/SiO_2 after wet etching Ni in $FeCl_3$ solution. Different layer thicknesses and different domains are clearly visible and their sizes are consistent with previous studies [3]. A statistical analysis of the grain size shows that ~35% of the sample is 4 layers thick with and average domain area of ~150 μm^2, see Figure S2c. Figure S2d shows an optical micrograph of a representative area of the same substrate after intercalation with $FeCl_3$, and the statistical analysis of this micrograph image shows no significant change of the average domain area upon intercalation which has to be expected since $FeCl_3$ is not etching graphene [1, 4 and 5], see Figure S2e.

Scanning electron microscope (SEM) images of Ni-CVD graphene on SiO_2 before and after $FeCl_3$ intercalation are shown in panels f) and g) respectively. After intercalation we observe a better contrast on the image, given by the higher conductivity of the graphene, and $FeCl_3$ residues (bright spots, Fe has a higher atomic number than carbon) on the surface and at the boundaries of the islands.

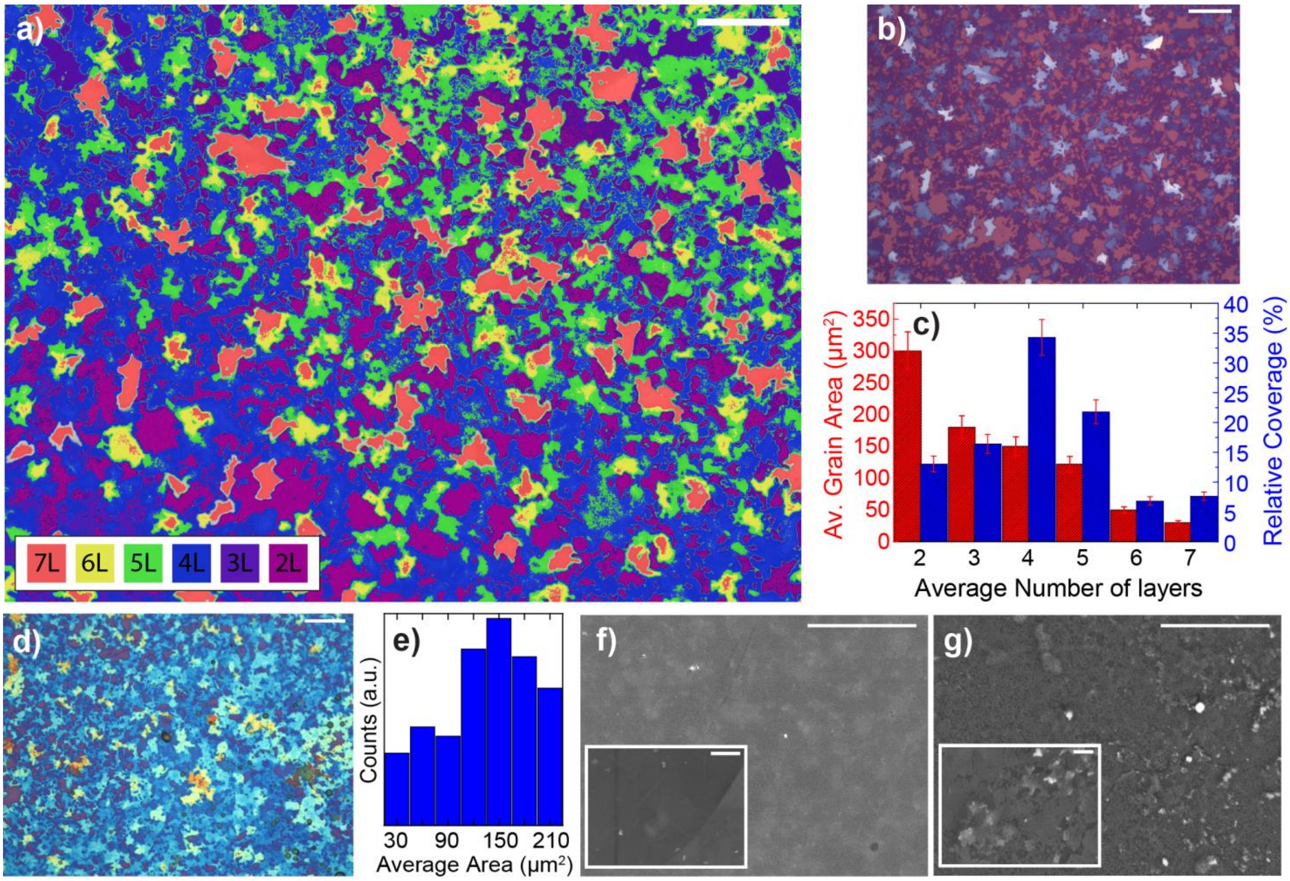

Figure S2. Characterization of Ni-grown CVD graphene transferred on Si/SiO_2 (with 300nm thick SiO_2). Panel a) shows a false-colour map of graphene grains masked according to the number of layers extrapolated from the optical micrograph in panel b). Panel c) shows a statistical study performed on the image in panel a) to determine the relative coverage and average grain size of the multilayer islands. Panel d) shows an optical micrograph of a representative area after $FeCl_3$ intercalation on which a statistical study to determine the average grain area has been performed, panel e). Panels f) and g) show SEM images of Ni-CVD graphene before and after $FeCl_3$ intercalation respectively. Scale-bars are 20μm in panels a), b), d) f) and g) and 2μm in the insets of panels f) and g).

3. **Comparison of the charge density estimated from Raman spectroscopy and from quantum oscillations in the magneto-conductance**

To assess the accuracy with which the stiffening of the E_{2g} phonon mode can be reliably used to estimate the charge density in $FeCl_3$-few-layer intercalated graphene ($FeCl_3$-FLG) we conduct a comparative study of the charge density obtained from the Raman G-peak shift and the period of the Shubnikov-de Haas oscillations (SdHO) presented in the work of ref. [1]. Figure S3 shows the Raman spectrum of $FeCl_3$-FLG and the G-peak shift. Using the theory in ref. [2] the G_2 peak at $G_2^{pos} = (1623.24 \pm 0.02) cm^{-1}$ gives a charge density of $n = (9.0 \pm 0.5) \cdot 10^{13} cm^{-2}$ and the SdHO measurements in ref. [1] report a value of $n = (10.700 \pm 0.005) \cdot 10^{13}\ cm^{-2}$. Hence the difference in charge density estimated from Raman and SdHO is $1 \cdot 10^{13} cm^{-2}$. This discrepancy can simply originate from the fact that Raman spectroscopy is a local probe (the laser spot-size is typically <1μm) whereas SdHO is probing the charge density on a macroscopic scale corresponding to the distance between source and drain contacts. Given the small discrepancy between the two measurements we can conclude that Raman spectroscopy is a valuable, non-destructive, tool to estimate the charge concentration in highly doped graphene, in particular over large areas.

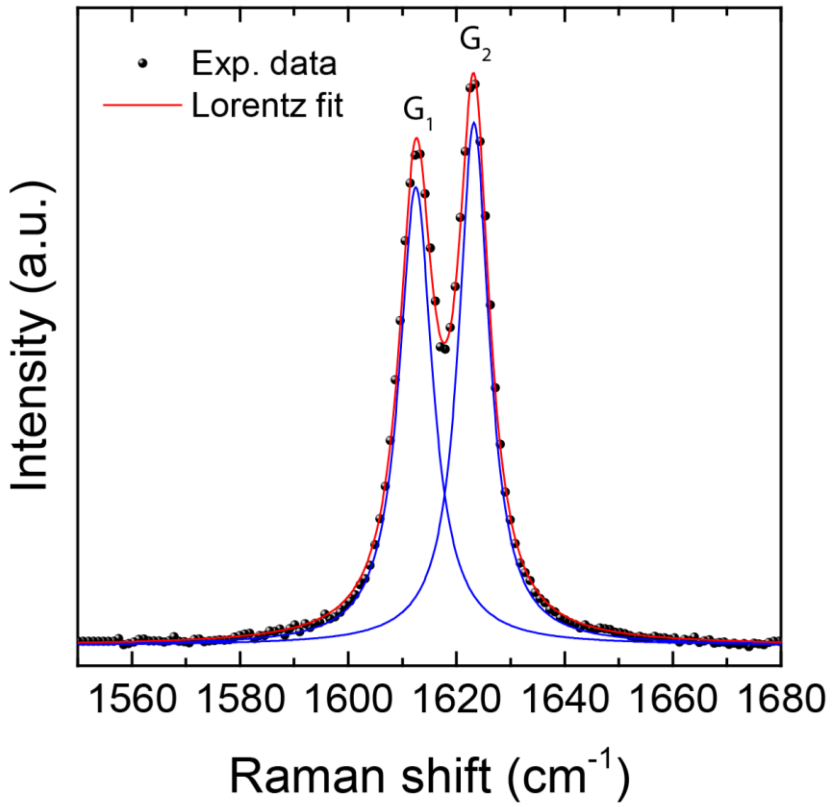

Figure S3. Raman spectrum of a $FeCl_3$-intercalated trilayer graphene taken from ref. [1] where stage-2 (G_1 peak) and stage-1 (G_2 peak) intercalation of graphene are present.

4. **Comparison of Raman maps of as-transferred Ni-CVD graphene and $FeCl_3$-intercalated Ni-CVD graphene.**

$FeCl_3$ is used at two separate steps in the fabrication. Firstly, the Ni substrate of the CVD-multilayer graphene is etched in 1 mol of $FeCl_3$ dissolved in de-ionised water. Secondly, the multilayer graphene transferred onto a glass substrate is exposed to vapours of $FeCl_3$ during the intercalation process. To elucidate the doping induced by the two separate exposures of the multilayer to $FeCl_3$ we present estimates of the charge density from the Raman measurements after each $FeCl_3$ exposure for a representative area of 100x100μm (Figure S4a). After the etching of Ni in $FeCl_3$ solution, we find experimentally an average doping of just $1 \cdot 10^{13} cm^{-2}$ and maximum values which do not exceed $2 \cdot 10^{13} cm^{-2}$, see Figure S4b. After intercalation of $FeCl_3$ the carrier concentration in the same area of multilayer graphene is as high as $5.5 \cdot 10^{13} cm^{-2}$ with an average value of $2.8 \cdot 10^{13} cm^{-2}$, see Fig.S4c. Indeed, the direct comparison of the Raman spectra before and after intercalation acquired at the same location clearly shows the shift of the G-peak, as expected for high charge density, see Figure S4d.

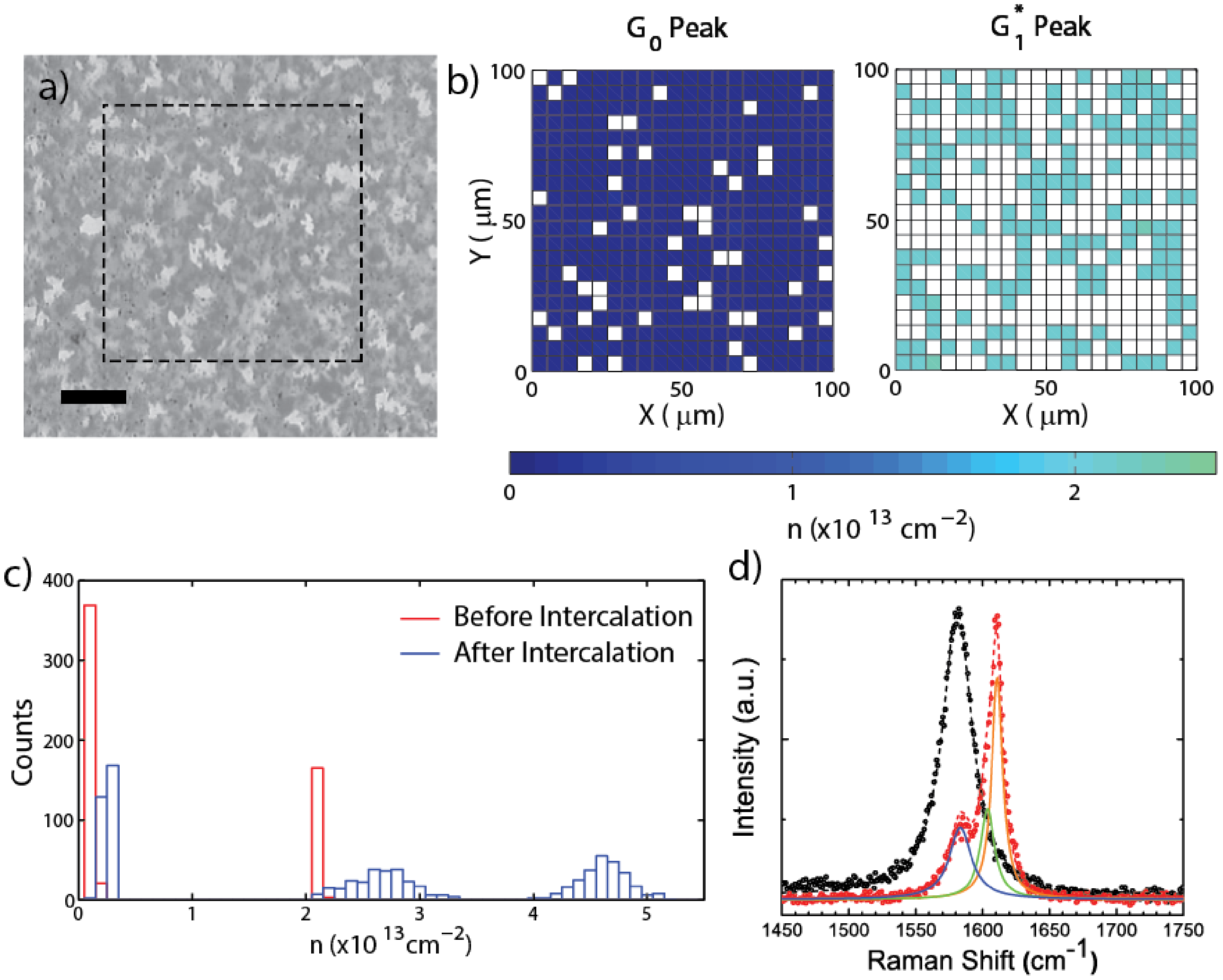

Figure S4: a) A grayscale optical microscope image of few-layer CVD graphene on glass (scale bar = 25μm). Carrier concentration maps over a 100x100um area (dashed line) are shown before, b), and after intercalation (main text Figure 2b). Panel c) shows a statistical analysis of the carrier concentration over the 100x100μm area highlighted in (a) before and after intercalation. d) Raman

spectra of the G peak at the same location before (black) and after (red) intercalation of few-layer graphene. Lorenztian fits of the G_0, G_1* and G_1 peaks are shown for the latter case.

5. **References**

[1] Khrapach, I.; Withers, F.; Bointon, T. H.; Polyushkin, D. K.; Barnes, W. L.; Russo, S.; Craciun, M. F.; *Adv. Mater*. **2012**, 24 2844.

[2] Lazzeri, M.; Mauri, F.; *Phys. Rev. Lett.* **2006**, 97, 266407.

[3] Kim, Keun Soo *et al.*; *Nature* **2009**, 457, 706-710.

[4] Bointon, T. H.; Krapach, I.; Yakimova, R.; Shytov, A. V.; Craciun, M. F.; Russo, S. *Nano Lett.* **2014**, 14, 1751.

[5] Wehenkel, D.J.; Bointon, T.H.; Peter, T.B.; Boggild, P.; Craciun, M.F.; Russo, S. *Sci. Rep.* **2015**, 5, 7609.
